# A Self-Applied Psychological Treatment for Gambling-Related Problems via The Internet: A Pilot, Feasibility Study

**DOI:** 10.1007/s10899-024-10318-2

**Published:** 2024-05-25

**Authors:** Laura Diaz-Sanahuja, Carlos Suso-Ribera, Ignacio Lucas, Susana Jiménez-Murcia, Cintia Tur, Patricia Gual-Montolio, Macarena Paredes-Mealla, Azucena García-Palacios, Juana María Bretón-López

**Affiliations:** 1 Department of Basic and Clinical Psychology and Psychobiology, Universitat Jaume I, Av. Vicent Sos Baynat, S/N, 12071 Castellón de La Plana, Spain; 2 CIBER de Fisiopatología de La Obesidad y Nutrición (CIBEROBN), Instituto de Salud Carlos III, Madrid, Spain; 3 Department of Clinical Psychology and Psychobiology, Universidad de Barcelona, Barcelona, Spain

**Keywords:** Gambling disorder, Ecological momentary intervention, Online treatment, Reach, Feasibility

## Abstract

The main purpose of this study was to evaluate the feasibility of an online psychological intervention for individuals with gambling-related problems, supported by ecological momentary assessments and interventions (EMAs and EMIs), along with weekly phone-calls, before conducting a randomized controlled trial. Participants were required to complete 3 of the 8 modules of the program based on cognitive-behavioral therapy (CBT) and extensions and innovations of CBT. The study measured the outcomes of feasibility (i.e., reach, appropriateness, technology literacy and technology usability, fidelity, and adherence). In terms of reach, 19.8% (*n* = 11) of the initial population met the inclusion criteria and completed the three modules (*mean age* = 41; 90.9% men). The perceived appropriateness and the technology usability after the first use were both excellent, fidelity and adherence to the online treatment (73.3%) were adequate. Adherence to the EMAs and the weekly phone calls were more modest (54.51% and 66.67%, respectively). The results of the present study show that an online treatment for gambling problems enhanced by EMA and EMI might be feasible but challenges were noted in terms of reach and adherence to these assessments and calls. These challenges are important to consider for future trials and the scalability of treatments for individuals with gambling disorders.

**Supplementary Information:**

The online version contains supplementary material available at 10.1007/s10899-024-10318-2.

## Introduction

Gambling disorder (GD) is a behavioral addiction included in the category of “Substance-Related and Addictive Disorders” of the Diagnostic and Statistical Manual of Mental Disorders (DSM-5). It is characterized by frequent preoccupations with gambling, craving, tolerance, repeated unsuccessful attempts to control or stop gambling, withdrawal symptoms (e.g., irritability or restlessness), gambling to escape from a dysphoric state, “chasing” losses, lying about gambling in significant relationships, and relying on others to finance gambling (APA, 2013). It is a persistent, recurrent pattern of gambling that is associated with substantial impairment (Potenza et al., 2019). For example, the suicide risk in this population is four times higher than in community samples (Wardle et al., 2020), which seems to be explained by certain psychological factors that are characteristic of individuals with GD, such as high impulsivity traits and difficulties regulating emotions (Mallorquí-Bagué et al., 2018). Throughout the paper, we will use the term “problem gambling” when individuals exhibit several symptoms of GD which correspond to 3 or 4 criteria, as well as the term “pathological gambling” when 5 or more criteria are met. These terms are generally used when referring to the Norc Diagnostic Screen for gambling problems (NODS; Becoña, 2004) outcomes.

The complexity of the symptomatology in individuals with GD is often accompanied by other psychological disorders, most frequently anxiety, mood disorders, and substance-use disorders (Cowlishaw et al., 2014; Lorains et al., 2011). It is estimated that the 1-year prevalence of GD oscillates between 0.12% and 5.8% globally (Calado & Griffiths, 2016). In Europe, the yearly prevalence of this disorder ranges from 0.1% to 3.4%. It has been argued, however, that these higher prevalence rates are due to infrequent consultations caused by low awareness of the illness in many patients. These patients often struggle to identify GD symptoms and its negative consequences, finding it difficult to recognize the importance of seeking treatment (Shah et al., 2020).

Encouragingly, there are evidence-based interventions to effectively manage GD. In particular, Cognitive Behavioral Therapy (CBT) is the most frequently used and evidence-based intervention to effectively treat GD, along with motivational interviewing (Menchon et al., 2018; Pfund, et al., 2020; Bodor et al., 2021; Tolchard, 2017). Cognitive restructuring, stimulus control, and exposure with response prevention are the most widely evidenced components of CBT in GD. However, emotional regulation has an essential role in GD and other behavioral addictions (Rogier & Velotti, 2018). Extensions and innovations of CBT have demonstrated their efficacy for substance and behavioural addictions, among which we find Mindfulness-based interventions (Maynard et al., 2018; Sagoe et al., 2021; Sancho et al., 2018); Dialectical and Behavioral Therapy (DBT) adaptations that include mindfulness, emotional regulation, interpersonal effectiveness, and distress tolerance modules that help acquire crucial skills to mitigate emotional dysregulation and maladaptive behaviours through purposeful actions aligned with specific goals and values (Cavicchioli et al., 2020); and interventions that draw upon principles of CBT, including mindfulness, and also on acceptance and positive psychology techniques to help reduce depressive symptoms and gambling-related issues that have shown moderate to strong effect sizes (Bücker et al., 2018). Mindfulness-based therapies enable individuals to not react automatically to emotions of negative or positive valence. For instance, in situations where discomfort arises, such as when experiencing cravings, which can affect one’s inhibitory control capability over gambling behavior (von Hammerstein et al., 2018). Consequently, both CBT and its extensions encompass essential therapeutic components to effectively address GD (Cavicchioli et al., 2020). However, despite the robustness of these interventions when addressing gambling problems, less than 10% of the individuals who suffer a GD seek help, a percentage that is significantly lower than that of other mental health conditions (Gainsbury, et al., 2014; Mojtabai et al., 2002; Suurvali et al., 2008). Also concerning, among those who receive treatment, dropout rates are often high, reaching up to 40% in face-to-face programs (Augner et al., 2022).

As noted earlier, an important barrier to treatment is that individuals with GD are often unwilling to admit that they have a problem and tend to minimize it (Suurvali et al., 2009). They generally seek help when these problems have become extremely severe and have a devastating impact on finances, interpersonal relationships, and physical and mental health (Evans & Delfabbro, 2005; Gainsbury et al., 2014). In addition to this impaired awareness of the problem, there are other barriers that may contribute to the challenges in seeking help. These can be internal, such as fear of stigma, shame, and denial, or external, such as the lack of available or easily accessible services, difficulties in attending treatment sessions due to geographical distance, lack of local expertise and resources, time constraints, and competing work and personal demands (Shah et al., 2020).

As already supported by some research, using Internet-based interventions could help improve access for individuals with GD to evidenced-based interventions. Goslar et al. (2017), for example, reported the effectiveness of two high-intensity structured web-based interventions when compared to face-to-face services for the reduction of problem gambling severity, gambling frequency, and financial loss at post-treatment (Carlbring & Smit, 2008; Casey et al., 2017). In addition, a recent meta-analysis showed that online psychological treatments for GD had moderate effects in the short-term (Augner et al., 2022), with significant positive pooled effect sizes (*g* = 0.41 for treatment–control comparison, and *g* = 1.28 for pre-post comparison). Online multi-session treatments also showed larger effects than brief interventions in reducing the amount of time and money spent on gambling (Peter et al., 2019). It is important to note that these self-guided treatments for GD have similar effectiveness when comparing interventions with or without therapist contact, but human contact shows additional advantages in terms of patient satisfaction (Goslar et al., 2017).

In sum, online interventions for individuals with GD appear to be an excellent alternative to make treatments more accessible and scalable. Attrition rates of Internet-based interventions, however, are still an important unresolved issue, with losses that oscillate between 6 and 65% (Bücker et al., 2018; Hodgins et al., 2019; Magnusson et al., 2019). Ecological Momentary assessment and interventions (EMA/EMI), as well as therapeutic support while the online interventions are carried out, are procedures that might help minimize attrition rates in online treatments (Díaz-Sanahuja et al., 2021). The literature on EMIs for individuals with GD is still scarce but encouraging. Hawker et al. (2021), for example, recently conducted a feasibility study with an EMI system to reduce the intensity of craving in people with gambling problems and showed reductions of 71% and 72% in the average number of gambling episodes and craving occurrences, an effectiveness rate that could potentially improve treatment adherence and satisfaction.

Given that there is still very little literature on the use of Internet treatments for individuals with GD enhanced with EMA/EMI, the objective of this study was to assess the feasibility of the “SIN JUGAR, GANAS” [YOU WIN BY NOT BETTING] program, a self-applied psychological online treatment for GD enhanced with EMA/EMI and supported by brief phone-calls. This feasibility, pilot trial will be crucial before conducting a larger-scale randomized controlled trial in terms of potential feasibility problems and preliminary efficacy, which is important for sample size estimation (Aschbrenner et al., 2022). All the previously mentioned aspects will be investigated over the first three treatment modules (i.e., motivation for change, psychoeducation and stimulus control, and responsible debt payment) to evaluate feasibility data before a full-length program is carried out.

## Method

### Study Design

The present research corresponds to a non-randomised pilot, feasibility study. It was approved by the Ethics Committee of the Universitat Jaume I (Castellón de la Plana; CD/026/2019) and was conducted following the international standards of the Declaration of Helsinki and good clinical practice.

### Participants, Recruitment, and Eligibility Criteria

To recruit participants, we disseminated the study through professional social networks (e.g., LinkedIn and the official website of the college of psychologists), as well as non-professional social networks (e.g., announcements on Facebook, Instagram, WhatsApp, and Twitter). We also contacted different associations and mental-health services focused on treating addiction. Leaflets and flyers were distributed at the university and nearby areas. Press advertisements and radio interviews were also conducted. Participants who were interested were contacted via e-mail (sinjugarganas@gmail.com) and received information about the study procedure. The recruitment process was conducted following the snowball recruitment technique. Table 1 shows the specific recruitment methods employed.

**Table 1:** Recruitment methods

Recruitment methods, Number of individuals who initially were contacted, 'total number of people recruited		
Paid announcements on Facebook by selecting the characteristics of the target population	4	0
Publications on Twitter by the ministry of health, the research group, and personal accounts	6	0
Publications on Instagram and Facebook by the Jaume I University, our research group account, and personal accounts	7	1
Publications on Facebook groups related to psychology and groups of pathological gamblers	80	42
WhatsApp groups	20	6
Newspapers	5	5
Radio interviews	3	0
Phone-calls to centres and associations related to the treatment of addictions	23	2

The inclusion criteria for the study included: being 18 years of age or older; having access to the Internet, a computer, an e-mail account, and having sufficient technological literacy to participate in the study. This means enough ability to use technology tools, which was assessed using a 5-point Likert scale: 0 “little to none”; 1″ low”; 2 “normal”; 3 “advanced”; 4 “expert level” (those with little to none or low were excluded); being able to understand, read, and write in Spanish; having a diagnosis of problem gambling or pathological gambling (scores from 3 to 10) in the Norc Diagnostic Screen for gambling problems (NODS; Becoña, 2004). Participants were excluded if they presented a high risk of suicide, a severe mental disorder, a medical illness that could interfere with the progress of the program, a moderate or severe substance dependence, if gambling occurred due to a manic episode, or if they were receiving another psychological treatment for gambling-related problems. To assess if participants met these inclusion/exclusion criteria, they first completed an online survey via Qualtrics and responded to sociodemographic and gambling severity questions. If they met the age and gambling severity criteria, the screening process continued, and the professional video-called the person to assess if the other inclusion criteria were also met. There was no financial compensation for participating in the study, and participants accepted the informed consent voluntarily.

### Intervention

“SIN JUGAR, GANAS” is an online psychological treatment for gambling-related problems based on cognitive-behavioral therapy and extensions and innovations of CBT such as mindfulness, emotion regulation strategies (e.g., opposite action technique and emotional distancing technique), distress tolerance strategies, and the establishment and planning of intentional behaviours towards concrete goals guided by personal values. The treatment is included in the www.psicologiaytecnologia.es website and consists of 8 therapeutic modules. In this pilot feasibility study, we included three out of the 8 modules: motivation for change, psychoeducation and stimulus control, and responsible debt return. Although it is suggested to complete one module per week, the duration of treatment needed for each individual is still unclear, taking into account the target population and the intervention format. In addition, even though the intervention includes a weekly telephone support call of around 10 min, it is also unknown whether this is feasible according to the characteristics of the target population. The protocol of the study by Díaz-Sanahuja et al. (2021) contains a more detailed explanation of the content and objectives of the modules, as well as the additional tools found in the web platform, such as home, calendar, plan for returning debts, my progress, and ‘What have I learned’? (See Supplementary material), and the use of an EMA/EMI. This EMA/EMI is an assessment carried out by participants using their smartphones through a Qualtrics link. Every day at 8 PM they receive a notification on WhatsApp to complete the assessment. It consists of four questions about their degree of gambling urges on a Likert scale from 0 (not at all) to 10 (maximum), as well as the frequency of gambling urges (never; sometimes; usually; many times, almost always), self-efficacy to cope with gambling urges on a Likert scale from 0 (not at all) to 10 (completely), and whether they have gambled or not. They are asked to respond based on what has happened in the last 24 h. Based on their answers, they are given a synchronous intervention that consists of a specific message more related to emotion validation and motivation for change. When they respond that they have gambled, in addition to this, they are provided with more questions related to a functional analysis of the lapse. For example, amount of money, time spent, location, thoughts, emotions, identifying what they think caused the lapse (e.g., whether motivation changed, if they minimized the negative consequences of gambling and maximized the positive ones, etc.), and finally, they are asked an open-ended question where they must indicate what they have learned from this lapse and what strategy they could apply in the future in a similar situation to avoid future lapses/relapses. It could help to be aware of the factors that could lead to the lapse and to motivate the patients to remain abstinent again, avoiding a relapse. This content is also considered in the weekly phone calls, however, the EMA/EMI is intended to help them at that specific moment. According to Trull and Ebner-Priemer (2009) and Shiffman (2009) although this EMA/EMI is not really “real-time” and relies on retrospection, the assessment occurs regarding very recent states or behaviors (on the same day) and enables the collection of repeated assessments over time in the natural environment and to deliver immediate intervention messages.

### Measures

#### Demographics, Screening, Diagnostic Measures, and Other Clinical Variables

The sociodemographic variables assessed were age, sex, marital status, type of coexistence, educational level, profession, occupational situation, income, country, and spiritual beliefs. In addition, we evaluated clinical variables. For example, we evaluated whether they had previously received psychological treatment for gambling problems or other reasons.

We assessed gambling severity using the NORC DSM-IV screening for gambling problems (NODS; Gerstein et al., 1999; Spanish version by Becoña, 2004). This is a hierarchically structured screening instrument for the assessment of gambling problems in the last 12 months. It has 17 dichotomous items. In our study, the mean NODS score was 9.73 (SD = 0.47, mode = 10, median = 10, range = 9—10). Of the 11 participants, 27.3% (n = 3) scored 9, and 72.7% (n = 8) scored 10, indicating that all of them were pathological gamblers. Internal consistency as measured by coefficient α was 0.51 for the NODS, which is weak (Tavakol & Dennick, 2011). Our study has a small sample size (n = 11). In addition, due to the characteristics of the questionnaire, which has a dichotomous response format, and the limited variability of the responses, given that all participants are clinical population, the Cronbach's alpha internal consistency index may not be accurate. However, Hodgins (2004) previously found a good reliability of the NODS, corresponding to an α of 0.79. In addition to gambling problems, we assessed their gambling history (Echeburúa & Báez, 1994), including the onset and aggravation of the gambling behavior and the main type of gambling behavior, as well as other gambling-related variables (e.g., economic debts, whether they have access to money, amount of time since the last bet, and risky places). We also evaluated possible comorbid diagnoses according to the Diagnostic and Statistical Manual of Mental Disorder (DSM-5), which were evaluated during a video-call of approximately one hour of duration. In addition, other clinical outcomes at pre-treatment are considered for the description of the participants’ profile such as the readiness to change and quality of life. The assessment tools used to evaluate these outcomes are The University of Rhode Island Change Assessment Scale (URICA) (Gómez-Peña et al., 2011; McConnaughy et al., 1983) and the quality of life index (QLI) (Mezzich et al., 1999, 2000).

The URICA assesses the pre-contemplation, contemplation, action, and maintenance stages of change proposed by Prochaska and DiClemente (1982), as well as the degree of ‘Readiness to change’ through 28 items rated on a 5-point Likert-type scale. Scores for each subscale are obtained by adding the corresponding items, which oscillate between 8 and 40. A global score of readiness to change is also calculated by adding the mean scores of the contemplation, action, and maintenance stages and subtracting the score obtained in the pre-contemplation stage. The total score can vary from -2 to + 14. The higher the value, the higher the readiness to change. A value lower than 8 means that the patient is in the pre-contemplation change stage, a score from 8 to 11 corresponds to the contemplation change stage, and a score of 12 or higher reflects an action change stage (DiClemente et al., 2004). The internal consistency of the overall score of ‘Readiness to change’ was adequate in the present study (*α* = 0.72).

The QLI measures quality of life using 10 dimensions that correspond to physical wellbeing, psychological/emotional well-being, self-care, independent functioning, occupational functioning, interpersonal functioning, social-emotional support, community and services support, personal fulfillment, spiritual fulfillment, and overall perception of quality of life. Items are rated on a 10-point Likert-type scale varying from 1 “poor” to 10 “excellent”. The overall score corresponds to the average of the item values and oscillates between 1 and 10. Scores from 1 to 4.5 indicate a perception of quality of life below the average. Values from 4.6 to 8.1 reflect average quality of life and scores of 8.2 to 10 reflect a perceived quality of life above the average. The internal consistency of the QLI in our sample was excellent (*α* = 0.89).

#### Primary Outcomes

The primary outcomes of this single-arm feasibility study were those of feasibility research (Arain et al., 2010; Lancaster et al., 2004; Whitehead et al., 2014):

- Reach. The percentage of participants who are willing to participate and the extent to which they are representative of the target population (Shaw et al., 2019).
- Treatment appropriateness, which refers to the perceived fit, relevance, compatibility, suitability, perceived usefulness, and practicability. Appropriateness was measured with the Treatment Expectations questionnaire (Borkovec & Nau, 1972), a self-report instrument that evaluates the participants' expectations about an intervention. It comprises 6 items referring to the extent to which the treatment is logical, the expected degree of satisfaction, the extent to which they would recommend it to others, the usefulness for their problem and for coping with other problems, and the actual aversiveness to use the program. The previous was evaluated using a Likert scale ranging from 0 “not at all” to 10 “very much”. The Spanish adaptation of the Treatment Expectations questionnaire has been used in previous research (Botella et al., 2016; Mira et al., 2019a, b, c; Tortella-Feliu et al., 2011).
- Usability and acceptability of the technology. System usability evaluates whether users can use the technology to achieve a particular goal in a given context. This was measured by the System Usability Scale (SUS) (Brooke, 1996; Castilla et al., 2023). The SUS assesses the usability of ICT applications using 10 items in which patients report the degree of agreement with a series of statements on a 5-point Likert scale (from 1 “Strongly disagree” to 5 “Strongly agree”). An overall score is obtained and is calculated as a percentage (0 − 100) considering a formula that consists of adding all the item values that range from 0 to 4 and multiplying the score by 2.5. The higher the percentage, the greater the perceived ease and product quality (Bangor et al., 2008). The Cronbach’s alpha of both the treatment expectancies scale and the SUS in our sample was good (0.88 and 0.85, respectively).
- Fidelity corresponds to the degree to which an intervention can be applied as initially intended. For instance, if the module time required, phone-call duration, treatment components delivered, and format applied are the same as planned.
- Adherence was evaluated as the number of days and minutes of platform use, the number of times that each module was reviewed, the percentage of daily evaluations completed, and the response rate and time spent on weekly-calls. In addition, the response rates to the daily assessment with EMA/EMI and the alarms triggered by gambling episodes were used to conduct a functional analysis.

#### Secondary Outcomes

Clinical variables measured at post-module, such as gambling severity, perceived self-efficacy to control gambling, and anxiety and depressive symptoms were included as secondary outcomes (preliminary effectiveness). We also investigated the utility of the EMAs, that is, the daily information provided by the patients regarding the number of gambling episodes, their duration (in minutes), and the money spent on gambling (in euros).

The gambling symptom assessment scale (G-SAS) (Kim et al., 2009). The G-SAS comprises 12 items rated on a 4-point scale and assesses gambling symptom severity in the past week. Specifically, it evaluates the pattern of change in subgroups of symptoms (e.g., gambling urges; average frequency, duration, and control of thoughts associated with gambling; time spent on gambling; anticipatory tension caused by an imminent gambling act; excitement associated with winning; emotional distress; and personal trouble). The total score is calculated by adding the different item scores and varies from 0 to 48. Scores from 8 to 20 represent mild severity; values from 21 to 30 reflect moderate symptom levels; scores from 31 to 40 reflect severe symptoms; and values from 41 to 48 indicate an extreme severity of gambling symptoms. This scale presented an excellent internal consistency in our sample (*α* = 0.96).

The Gambling self-efficacy questionnaire (GSEQ) (May et al., 2003; Winfree et al., 2013). The GSEQ evaluates the perceived self-efficacy to control gambling in risky situations associated with intrapersonal factors such as (un)pleasant emotions or gambling urges, as well as with interpersonal factors, such as social pressure or conflicts (Marlatt, 1985). This scale contains 16 items rated on a 6-point Likert scale ranging from 0% (Not at all confident) to 100% (Very confident). The overall score is obtained by calculating the mean response on the items and varies from 0 to 100, with higher overall scores indicating higher confidence about controlling one’s gambling behavior. The Cronbach’s alpha of the GSEQ in our sample was excellent (*α* = 0.95).

Overall depression severity and impairment scale (ODSIS) (Bentley et al., 2014; Mira, González-Robles et al., 2019). The ODSIS evaluates the severity and functional impairment associated with depression during the past week through five items rated on a 5-point Likert scale that varies from 0 to 4. The overall score is calculated by adding the values of the items and oscillates between 0 and 20. Scores of 5 or higher indicate the presence of depressive symptoms. The Cronbach’s alpha of the ODSIS in our sample was excellent (*α* = 0.91).

The overall anxiety severity and impairment scale (OASIS) (Campbell-Sills et al., 2009; González-Robles et al., 2018). The OASIS is a 5-item self-report instrument that measures one factor defined as the severity and frequency of anxiety symptoms, behavioral avoidance, and functional impairment in the past week. It is assessed on a 5-point Likert scale ranging from 0 to 4. The overall score is obtained by adding the values of the items and varies between 0 and 20. Scores higher than 8 demonstrate the presence of significant anxiety symptoms. The Cronbach’s alpha of the OASIS in the present study was below the threshold required for good internal consistency *(α* = 0.68) (Tavakol & Dennick, 2011). These results should be considered with caution as the small sample size may be making the estimation of internal consistency less precise. However, there is no need for concern because this instrument was validated in Spanish in people suffering from mood disorders (*n* = 583) and showed an adequate Cronbach's alpha (*α* = 0.86) (González-Robles et al., 2018).

Table 2 shows the assessment instruments used.

**Table 2:** Assessment instruments and the different time frames used

Measures	Screening	Pre-Treatment	Daily	Post-Module
Sociodemographic data	X			
NODS (12 months)	X			
GI	X			
URICA		X		
QLI		X		
G-SAS (gambling urges)		X		X
GSEQ		X		X
OASIS				X
ODSIS				X
EMA measures (gambling episodes and duration and money spent)			X	
Technological profile		X		
Treatment expectations questionnaire		X		
SUS				X*

DERS: Difficulties in Emotion Regulation Scale; DM: Daily Measure: GI: Gambling history interview and current gambling situation and related variables assessment; GRCS-S: Gambling-Related Cognitions Scale; G-SAS: The Gambling Symptom Assessment Scale; GSEQ: Gambling Self-Efficacy Questionnaire; HADS: Hospital Anxiety and Depression Scale; NODS: NORC DSM-IV Screen for Gambling Problems; OASIS: The Overall Anxiety Severity and Impairment Scale; ODSIS: The Overall Depression Severity and Impairment Scale; PANAS: The Positive and Negative Affect Schedule; QLI: Quality of Life Index; SUS: System Usability Scale; UPPS-P: The Short UPPS-P Impulsivity Scale; URICA: The University of Rhode Island Change Assessment Scale

* After the first use

### Statistical Analysis

First, descriptive statistics (frequency, mean, and standard deviation) were conducted on the sociodemographic and clinical characteristics of the sample. The percentage of participants willing to participate assessed for eligibility was analysed and the representativeness of the sample was considered based on the descriptive statistics mentioned (reach). Treatment adherence was evaluated considering the number of days the platform was used for each module, the average duration of platform use in minutes, and the number of times each module was reviewed. EMA adherence was calculated as the percentage of daily evaluations completed. Adherence to the weekly phone calls was also calculated. Treatment appropriateness and usability were evaluated after the first use of the web-platform when the welcome module ended. Non-parametric analyses, including paired samples Wilcoxon tests were carried out to evaluate the preliminary effectiveness of the intervention enhanced with EMA/EMI. Specifically, we calculated changes in gambling urges (measured by the first four items of the GSAS) and gambling self-efficacy (GSEQ questionnaire) from pre-treatment to post-module 3, as well as changes in anxiety (OASIS questionnaire) and depressive symptoms (ODSIS questionnaire) from post-module 1 to 3. Statistical analyses were performed using the IBM SPSS Statistics program version 28.

## Results

### Participant Flow and Reach

**Fig. 1:**
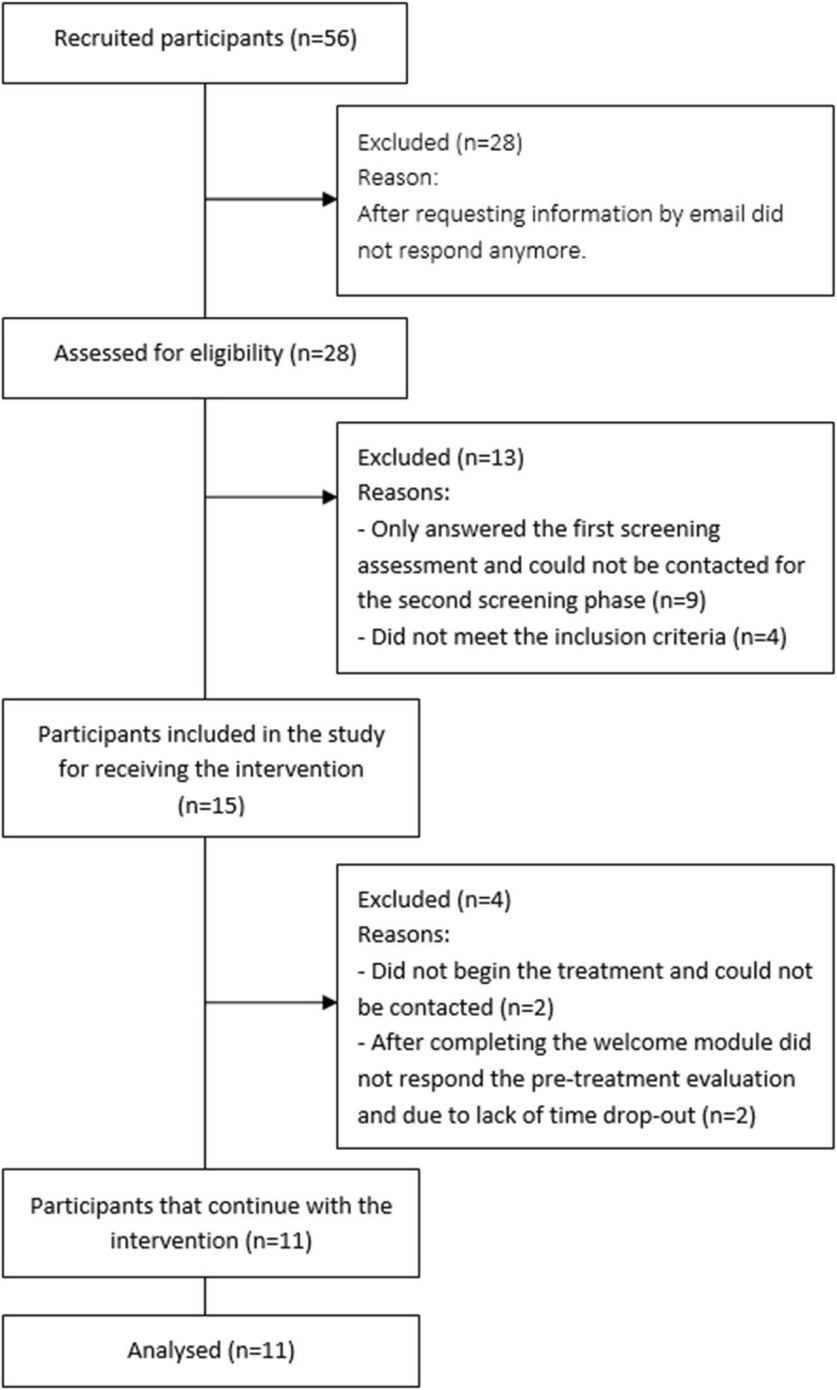
Flow diagram of the study

The flow diagram (Fig. 1) shows the flow of the participants’ recruitment. Initially, 56 people were interested in the study. Of these, 50% (*n* = 28) did not complete the initial survey for the assessment of their inclusion/exclusion criteria after receiving the information and the remaining 28 were assessed for eligibility. Nine of them only answered the first screening assessment (NODS and sociodemographic data) but did not attend the appointment to assess their gambling history and gambling-related variables, as well as possible comorbidity, and were therefore excluded. In addition, 4 of them were excluded after the full screening evaluation: one participant did not meet the inclusion criteria of gambling severity, one did not meet the criteria of age, one participant was excluded because he presented high suicidal tendencies, and one presented comorbidity with severe mental health disorders. These participants were offered alternative treatments in a blended format with more intense therapeutic support. No participant was excluded due to low technological literacy: 72.7% (*n* = 8) reported a score value of 2 which means “normal” and that they consider themselves capable of doing what was needed, while 27.3% (*n* = 3) reported a score of 3, which corresponds to an advanced level, so they consider that they know how to do more things than other people.

Finally, 26.8% (*n* = 15) of the initial sample was included in the study. Of those, 4 withdrew from the intervention. Specifically, 2 did not begin the treatment and could not be contacted and 2 participants quit after completing the welcome module and did not respond to the pre-treatment evaluation because of lack of time. Consequently, the final sample used in this study included 11 participants who had completed the first three modules.

### Participant’s Sociodemographic, Gambling History, and Other Related and Clinical Characteristics at Pre-Treatment

The vast majority of the participants were male (90.9%; *n* = 10) with a mean age of 41 years (*SD* = 13), ranging from 26 to 68 years. Participants were Spanish-speakers, with 54.5% (*n* = 6) from Spain, 36.4% (*n* = 4) from Mexico, and 9.1% (*n* = 1) from Colombia. Most were married or in a relationship (81.9%). Only 18.2% were single. Regarding educational level, most of them had completed higher education (81.8%). The remaining participants (18.2%) had only completed elementary education. Regarding employment status, 63.6% (*n* = 7) were employed. The remaining participants were unemployed (9.1%; *n* = 1), on temporary leave (9.1%;* n* = 1), on long-term sick leave (9.1%; *n* = 1), or retired (9.1%; *n* = 1). Professions were mainly related to the tertiary sector (54.5%;* n* = 6) (e.g., education) and the secondary sector (36.4%; *n* = 4) (e.g., industry and construction). Only 1 participant worked in the primary sector (e.g., agriculture). The average net income per year was 24,696.65€ (*SD* = 10,795.03), ranging from 3,570.07 € to 41,754.13 €. Most participants were not religious/spiritual (*n* = 9; 81.9%). Half of them (*n* = 5; 54.5%) had previously sought help for gambling problems and 27.3% (*n* = 3) for other reasons (e.g., anxiety and depressive symptoms; see Table 3).

**Table 3:** Participants’ sociodemographic data and history of psychotherapy

Gender, *n* (%)	
Male	10 (90.9)
Female	1 (9.1)
Age, mean (*SD*)	41 (13)
Marital status,* n* (%)	
Married/in a relationship	9 (81.9)
Single	2 (18.2)
Educational level completed, *n* (%)	
Elementary education	2 (18.2)
Higher education	9 (81.8)
Employment status, *n* (%)	
Employed	7 (63.6)
Unemployed	1 (9.1)
On temporary leave	1 (9.1)
On long-term sick leave	1 (9.1)
Retired	1 (9.1)
*Net incomes per year*, mean* (*SD*)	24,696.65 (10,795.03)
Religious/spiritual beliefs,* n* (%)	
None or slightly	9 (81.9)
Very much	2 (18.2)
Previous psychological assistance for GD, *n* (%)	5 (54.5)
Previous psychological assistance for other reasons, *n* (%)	3 (27.3)

*Data adjusted by country (Colombia, Mexico, USA, and Spain) considering Purchasing power parities (PPP) currency conversion rates that attempt to equalize the purchasing power of different currencies, eliminating differences in price levels between countries

The mean age of onset of the gambling behavior was 26.18 years (*SD* = 8.51), ranging from 10 to 38. The mean age at which the participants perceived gambling behavior to be problematic was 30.27 years (*SD* = 9.57), ranging from 18 to 52 years. Most participants did not have a family history of problem gambling (*n* = 7; 63.6%) or substance-use disorders (*n* = 9; 81.8%). However, 4 (36.4%) of them had a family history of gambling problems and 2 (18.2%) exhibited substance-use addictions. The main gambling behavior types were sports betting (*n* = 5; 45.5%) and slot machines (*n* = 4; 36.4%). Poker (*n* = 1; 9.1%) and roulette (*n* = 1; 9.1%) were less frequent. The most common gambling format was online only (*n* = 5; 45.5%), followed by onsite only (*n* = 3; 27.3%), and a combination of both online and onsite (*n* = 3; 27.3%). Nearly all participants had financial debts before starting the intervention (*n* = 10; 90.9%). Among those with debts, the average amount owed was 19,790.07 € (*SD* = 19,782.85), ranging from 816.02 € to 61,457.70 €. The average number of days without gambling at pre-treatment was 14.4 (*SD* = 16.73), ranging from 0 to 45 days. Gambling history and other related variables are summarized in Table 4.

**Table 4:** Gambling history and other related variables at pre-treatment

Age of onset of gambling behavior*, mean* (*SD*)	26.18 (8.51)
Age of perceiving gambling as problematic behavior, *mean (SD*)	30.27 (9.57)
Family history of problem gambling, *n* (%)	
Yes	4 (36.4)
No	7 (63.6)
Family history of substance-use disorders, *n* (%)	
Yes	2 (18.2)
No	9 (81.8)
Main gambling behavior, *n* (%)	
Sports betting	5 (45.5)
Slot machines	4 (36.4)
Poker	1 (9.1)
Roulette	1 (9.1)
Gambling format, *n* (%)	
Land-based	3 (27.3)
Online	5 (45.5)
Both	3 (27.3)
Economical debts, *n* (%)	
Yes	10 (90.9)
No	1 (9.1)
*Amount of debts (€), *mean* (*SD*)	19,790.07 (19,782.85)
Number of days without gambling *mean* (*SD*)	14.4 (16.73)

* Data adjusted by country (Colombia, Mexico, USA, and Spain) considering Purchasing power parities (PPP) currency conversion rates that attempt to equalize the purchasing power of different currencies, eliminating differences in price levels between countries

All the participants included in the study suffered from GD. The participants' average gambling severity (NODS questionnaire) in the past 12 months was 9.7 (*SD* = 0.47), ranging from 9 to 10. Three participants (27.3%) were diagnosed with GD only, while the remaining participants (*n* = 8; 72.7%) exhibited comorbidity with other psychological disorders. Among these, three participants (27.3%) had one additional disorder, three (27.3%) had two comorbid disorders, and three (27.3%) had three. The most common comorbid disorders were major depressive disorder (*n* = 7), low risk of suicidality (*n* = 5), panic disorder (*n* = 2), alcohol use disorder (*n* = 2), generalized anxiety disorder (*n* = 1), binge-eating disorder (*n* = 1), and substance use disorder (cocaine) (*n* = 1). Most of the participants experienced anxiety symptoms alongside their GD (63.6%; *n* = 7), (HADS questionnaire), but did not take medication for anxiety symptoms (*n* = 10; 90.9%).

Before beginning the intervention, 10 participants (90.9%) were in the stage of preparation/action (URICA questionnaire) and presented a low perceived ability to deal with gambling urges when they faced risky situations (GSEQ questionnaire). They also reported that their gambling had an impact on their psychological/emotional quality of life (QLI questionnaire). The descriptive information (i.e., means, standard deviations, and ranges) for the clinical variables and quality of life is shown in Table 5.

**Table 5:** Mean, standard deviation (SD), and range of the clinical variables and quality of life

	Mean	SD	Range
Gambling severity			
NODS (past year)	9.7	0.47	9 - 10
GSAS total score (past week)	20.36	12.96	3 - 44
Gambling impulsivity (GSAS)	6.45	4.76	0 - 16
Readiness to change (URICA)	12.1	1.3	9.4 - 13.7
Gambling self-efficacy (GSEQ)	38.4	24.2	15 - 100
Quality of life (QLI)	6.1	1.7	4.3 - 9.4

### Other Feasibility Outcomes

#### Treatment Appropriateness

**Fig. 2:**
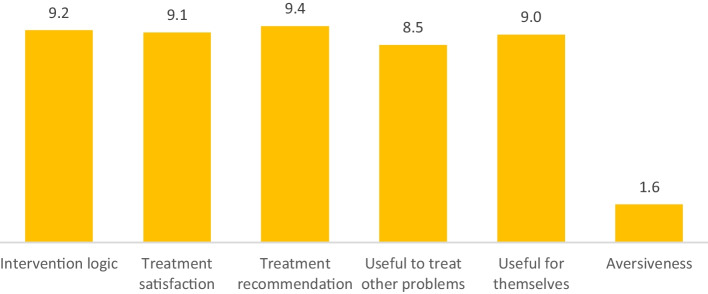
Mean scores of the treatment expectations scale items

According to the treatment expectations questionnaire (Borkovec & Nau, 1972), the participants showed high expectations towards the treatment before beginning the intervention (i.e., anticipated appropriateness). The mean score obtained was 52.55 (*SD* = 7.69) on a scale ranging from 36 to 60. The treatment was found to be logical (*M* = 9.2; *SD* = 1.3), potentially satisfactory (*M* = 9.1; *SD* = 1.3), likely to be recommended to others (*M* = 9.4; *SD* = 1.2), useful for the patient’s problem (*M* = 9; *SD* = 1.3), useful in treating other problems (*M* = 8.5; *SD* = 1.6), and non-invasive (*M* = 1.6; *SD* = 2.5). Scores ranged from 6 to 10 in all the items except in the aversiveness item, which varies from 0 to 7 and is the only item in which lower scores are preferred. These results are shown in Fig. 2.

#### System Usability and Acceptability

In terms of the system usability after the first use of the online platform (SUS; Brooke, 1996), the global score corresponds to a percentage and can range from 0 to 100. Results showed a mean of 83.6 (*SD* = 15.5; *range* = 57.5- 100) in the present study. According to the qualitative scale developed by Bangor et al. (2008), this means that the perceived system usability ranged from okay to the best imaginable, but would, on average, correspond to “Excellent”. Three participants (27.3%) considered it “best imaginable”, another three (27.3%) said usability was “excellent”, two (18.2%) considered it to be “good”, and three (27.3%) qualified it as “okay”. None of the participants considered the usability to be “poor”.

#### Adherence to the Web Platform, the Phone Calls, the EMA/EMI Tool, and Fidelity

Regarding the adherence to the use of the platform, this was adequate. Specifically, 73.3% of participants who were included in the study completed the first three modules. Table 6 shows the *means* (*SD*) and *ranges* of the number of days the participants accessed the platform, the duration (minutes) to complete each module, and the number of times each module was reviewed. The module that took the longest time to complete was module 2 (psychoeducation), followed by module 3 (stimulus control), module 1 (motivation for change), and finally the welcome module. Despite the recommendation to carry out one module per week, finishing modules 2 and 3 required more time, approximately a mean of two weeks. In addition, regarding the possibility of reviewing the modules, it was generally infrequent.

**Table 6:** Mean, standard deviations (SD), and range of the online platform usage up to module 3

	GWM		SWM		M1		M2		M3	
	*M (SD)*	*Range*	*M (SD)*	*Range*	*M(SD)*	*Range*	*M (SD)*	*Range*	*M (SD)*	*Range*
Days used	2.45 (2.21)	1–8	1.18 (.40)	1–2	5 (5.62)	1–16	14.91 (20.19)	1–71	12.71 (11.10)	1–30
Duration (minutes)	378.55 (119.23)	249–640	239.36 (51.16)	134–323	537.2 (148.4)	316–743	896.73 (407.96)	457–1542	535 (263.77)	356–1085
Times reviewed	0.55 (1.04)	0–3	0.73 (1.27)	0–4	0.27 (.65)	0–2	0.91 (.30)	0–1	0.57 (1.13)	0–3

GWM: General Welcome Module; M: Mean; M1: Module 1; M2: Module 2; M3: Module 3; r: Range; SD: Standard deviation; SWM: Specific Welcome Module

The ratio between the compliance time for each module and the prescription time was calculated. The prescription time per module is variable given that each module has a certain number of pages. Approximately a maximum of 15 min per page was calculated. Considering this and the estimated time per module, we observed that the participants needed approximately 2 to 3 times more than the established length of time (minutes). Regarding the number of days used to complete the modules, it was observed that to complete the welcome and motivation for change modules, they required fewer days than prescribed. As for the psychoeducation module (module 2) and the module on stimulus control and responsible debt return (module 3), a greater number of days were used, approximately twice as many. These data can be seen in detail in Table 7.

**Table 7:** Ratios adherence to the online platform usage up to module 3

Modules	CT	No pages	PT	CD	PD	R (minutes)	R (days)
GWM	378.55	11	165	3.63	7	2.29	0.35
SWM	239.36	9	135	5	7	1.77	0.17
M1	537.20	14	210	14.91	7	2.56	0.71
M2	896.73	19	285	12.71	7	3.15	2.13
M3	535	14	210	3.63	7	2.55	1.81

CD: Compliance days; CT: Compliance time (minutes); GWM: General Welcome Module; M1: Module 1; M2: Module 2; M3: Module 3; No pages: number of pages; PD: Prescription days; PT: Prescription time (minutes); R: Ratio (compliance minutes or days/ prescription minutes or days); SWM: Specific Welcome Module

**Fig. 3:**
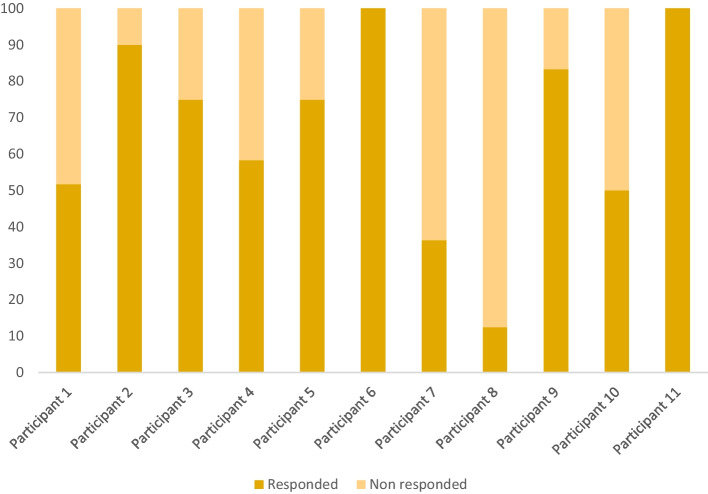
Response rates to the weekly phone calls per participant

The percentage of answered phone-calls was 66.57% (*SD* = 27.58), which ranged from 12.5% to 100% across participants (see Fig. 3). The average duration of the phone calls was 11.23 min (*SD* = 6.32), ranging from 5.3 to 24.75 min. *Means*, *SD*, and the *range* of the duration of phone calls per participant are shown in Table 8.

**Table 8:** Duration of the weekly support phone call

	*Mean*	*SD*	*Range*
Participant 1	5.73	3.55	3–17
Participant 2	6.00	2.18	3–10
Participant 3	7.20	2.05	5–9
Participant 4	11.40	9.44	1–30
Participant 5	5.33	1.15	4–6
Participant 6	13.29	3.45	9–18
Participant 7	24.75	24.39	9–61
Participant 8	9.00	2.65	6–11
Participant 9	15.00	3.08	10–17
Participant 10	6.66	1.15	6–8
Participant 11	19.20	8.84	11–29

**Fig. 4:**
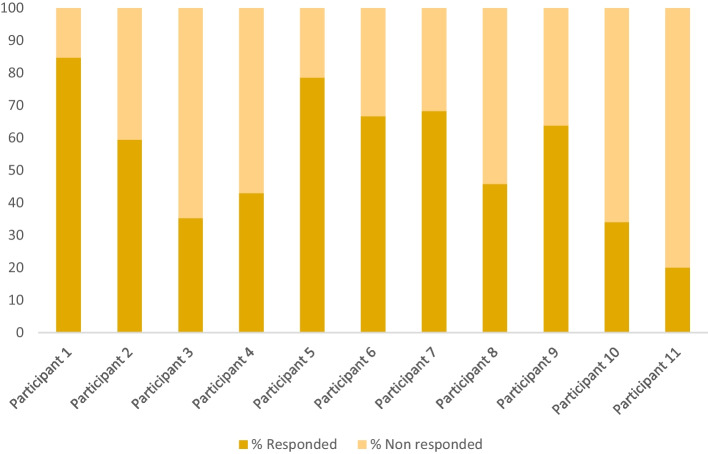
Response rates to the daily EMA/EMI per participant

The response rate to the daily assessment with the EMA/EMI was 54.51% (*SD* = 20.31; *range* = 20% -84.73%). Response rates per participant are shown in Fig. 4.

Regarding fidelity, there were not changes or adaptations to the program’s administration format, the amount of modules presented, or the type of support. Nevertheless, we were more flexible with the amount of time allowed for each module and the duration of the phone-calls.

### Preliminary Effectiveness Data

#### Progress of the Post-Module Outcomes

Gambling urges measured by items 1 to 4 of the GSAS (Kim et al., 2009), which could range from 0 to 16, decreased from pre-treatment (*Mdn* = 7) to post-module 3 (*Mdn* = 2) and a Wilcoxon test indicated that the differences were significant (*T* = 1.5,* z* = -2.12,* p* = 0.03). Gambling self-efficacy to cope with gambling urges also improved (i.e., increased) from pre-treatment (*Mdn* = 36.25) to post-module 3 (*Mdn* = 71.88) and the differences were also significant (*T* = 26, *z* = -2.03,* p* = 0.04).

Improvements in gambling urges and perceived gambling self-efficacy were accompanied by a slight decrease in anxiety and depressive symptoms from post-module 1 *(Mdn* = 5 in both outcomes) to post-module 3 (*Mdn* = 4.5 in both outcomes). However, a Wilcoxon test indicated these differences were not significant for anxiety (*T* = 8, *z* = -0.54, *p* = 0.59) or for depressive symptoms (*T* = 7.5, *z* = -0.63,* p* = 0.52).

#### Utility of the EMA

Thanks to the EMA, we obtained daily information about the number of gambling episodes experienced during the day, their duration, and the money spent on gambling. Over the three modules, five participants (45.5%) informed of at least one gambling episode. Of these, the mean number of gambling episodes (relapses) was 2 (*SD* = 1.41), ranging from 1 to 4. The average total amount of money spent on gambling by the five participants who reported relapses was 351.11 € (*SD* = 388.74), ranging from 71.53 to 1020.02 € adjusted by country and local economic standards. Finally, the average duration of all gambling episodes of these five participants was 197.71 min (*SD* = 136.62), varying from 45.25 to 392.33 min across individuals.

## Discussion

This study aimed to show preliminary data about the feasibility (i.e., reach, appropriateness, technology usability, fidelity, and adherence) of the “SIN JUGAR, GANAS” program for inndividuals dealing with gambling-related problems. The study was carried out before conducting a randomized controlled trial to investigate feasibility issues and to describe preliminary effectiveness data of the participants’ progress in managing gambling urges, gambling self-efficacy, anxiety levels, and depressive symptoms. These variables were measured using the web-platform after each module and daily EMA usage. Overall, the feasibility results were encouraging, except for the program’s reach, and preliminary effectiveness findings indicate some improvements, especially regarding the severity of gambling symptoms and self-efficacy to deal with gambling. However, the results were more modest for anxiety and depression.

In terms of reach, 50% of the people who requested information were willing to participate. However, of these, only 19.8% *(n* = 11) continued and completed the three modules. Even though we used a broad spectrum of dissemination strategies (e.g., professional and non-professional social networks, press and radio, health centers, gambling-related organizations, and associations), reach was problematic. One finding from this study was that while some non-digital strategies were used, the majority of potential participants were mainly found through online channels. This highlights the limitation of solely depending on the Internet for recruitment. It is important to have access to local associations and services that focus on addiction treatement.

The sociodemographic and clinical profile of the sample mainly consisted of men (90.9%), with a mean age of 41 years, married or in a relationship, who had completed higher education level studies, and were employed. All the participants were pathological gamblers according to the NODS (Becoña, 2004; Gerstein et al., 1999), which assesses gambling symptomatology during the previous 12 months. Although they were involved in gambling activities since the mean age of 26, they perceived it as a problematic behavior at the mean age of 30 years, after having approximately four years of gambling history. The main forms of gambling behavior were sports betting and slot machines, either online format or in combination with land-based formats. There was a lower percentage of participants gambling only in an onsite format. Most of them (90.9%) reported having financial debts. Our results are in line with previous literature, which supports the representativeness of the sample obtained with our recruitment procedures–which would be positive for reach purposes. The sociodemographic characteristics of our sample are similar to those of Aragay et al. (2021), in which the overall results indicated that participants had a mean age of 45 years, 94.3% were men, they generally had a stable partner, were employed, and indicated an age of gambling onset of approximately 26 years and a gambling history of approximately 5 years. Regarding the type of game and its modality, the most common gambling modes in 2019 in Spain were online sports-betting (31%) and land-based slot-machines (21%) (Dirección General de Ordenación del Juego, 2019; Jiménez‐Murcia et al., 2014), which is again consistent with our sample characteristics.

Online gambling gaining popularity could increase the risk of developing a GD due to its accessibility and the availability of different types of online games (e.g., sports betting, poker, casino games, bingo, and gambling machines) (Aragay et al., 2021; Chóliz, 2016). Sports betting is one of the most prevalent types of game, along with slot machines, with a tendency towards its online format. In our study, most of the participants reported having completed university studies. However, past research examining the profile of participants involved in different types of games found that they typically had only completed primary or secondary education (Aragay et al., 2021). There are probably different types of profiles depending on the type of game participants are involved in. In particular, individuals who bet on sports are more likely to be younger, single, with higher education, and have higher incomes compared to other types of gamblers (Cooper et al., 2022; Dowling et al., 2017; Jiménez-Murcia et al., 2021; Subramaniam et al., 2015). Aragay et al. (2021) also found this specific profile when analyzing only the group of sports betters compared to the group who wagered on land-based slot machines.

In terms of the clinical outcomes profile, our results are similar to previous findings (Zhang et al., 2018), in which approximately 98% of participants suffered from a GD. Several studies indicated that gamblers often have difficulties recognizing their gambling problems (Suurvali et al., 2009) and a high percentage of gamblers sought help when gambling severity was already very high and there was a high impairment or interference in their quality of life (Petry et al., 2005). Aragay et al. (2021) indicated that the overall duration of the GD before the treatment initiation, including online sports betting and land-based slot-machines gamblers, is over five years. Considering only the sports betting gamblers, this period was shorter, which points to different profiles according to the type of gambling behavior. Thus, the sample is representative of the target population according to previous literature (Shaw et al., 2019).

Recruitment and reach difficulties could be influenced by this tendency to ignore the gambling problem until it is very severe. People often seek help when they are in the action stage of readiness to change and experiencing severe symptomatology. This is consistent with our findings, as the sample of the current study consisted of people with GD who were mostly in the action stage. The problem awareness could be associated with the fact that gambling is an acceptable and normalized leisure activity, a means for feeling pleasure and gratification, and GD is defined as a more ego-syntonic disorder (el-Guebaly et al., 2012). For instance, regarding sports-betting activities, there is an established relationship between fun, sports, competition, friendship, and other values associated with youth (Aragay et al., 2021), which probably makes it difficult to recognize when there is problematic gambling (Bijker et al., 2022).

In terms of appropriateness and treatment valuation, the participants had high expectations before starting the intervention. They considered the treatment as logic, they perceived that it would satisfy them, they indicated that they would recommend it to others, they mentioned that it would be useful for the patient’s problem and other problems, and the perceived aversiveness was generally low. These results are in line with other well-established works on the use of online treatments for depression (Romero et al., 2019), which are encouraging the present and future Internet-based treatments for GD.

Regarding fidelity, it was generally not necessary to carry out adaptations on the program’s administration format, the number of modules presented, or the type of support, but we were more flexible with the amount of time allowed for each module and for the duration of the phone-calls. We recommended completing one module per week. The mean duration per module ranged from 4 to 15 h. However, the participants needed a mean of three days to complete the welcome module and the pre-treatment assessment, a mean of five days for module 1 (motivation for change), 15 days for module 2 (psychoeducation), and 13 days for module 3 (stimulus control). Thus, the psychoeducation and stimulus control module took longer than expected. In general, the participants needed about twice as much time as prescribed. However, although they required more time, the welcome and motivation for change module could be completed in less than a week. However, the psychoeducation and stimulation control and responsible debt return modules did need twice as many days to complete. While some previous work did not indicate this need to increase the duration of Internet treatment for GD (Carlbring & Smith, 2008; Myrseth et al., 2013), more research is needed in this area to explore which contexts, for which programs, or for which participants it is important to be flexible with the duration of Internet interventions for GD. In other conditions, for example, emotional disorders, we already have examples of studies recommending the completion of modules over longer periods (i.e., approximately every two weeks) (Mira et al., 2019c), so flexibility might be recommended.

The average adherence to phone-calls was 66.67% and their mean duration was 11.23 min, ranging from 5 to 25 min. This duration was also longer than planned, which affects fidelity. Phone calls took longer when participants presented lapses because the therapist had to make more effort to motivate the participants to continue with the treatment and to avoid gambling again. The duration of phone calls in previous studies regarding GD treatment that included therapist support ranged from 15 to 45 min, so the reported results in the current study are in accordance with previous literature (Carlbring & Smith, 2008; Castren et al., 2013; Myrseth et al., 2013).

Treatment adherence is an important issue to address because treatment dropout rates are high in internet interventions (Pfund et al., 2021). Although the results regarding the contribution of this therapeutic support for self-guided interventions needs more research, some studies report evidence that therapeutic support (e.g., via e-mails, phone-calls, or other channels during therapy) could have a better impact (Petry et al., 2017; Rash & Petry, 2014; Sagoe et al., 2021). Even though the response rates to the phone calls were not always satisfactory, the overall results would support their inclusion in future studies.

In addition to the adherence to the treatment, the adherence to the daily EMA/EMI responses was also modest (i.e., 54.51% of responses provided). These findings are in the same line as Hawker et al. (2021), who found compliance rates for EMA of 51% and EMI of 15%. This suggests that daily evaluation has to be improved, maybe with gamification elements and a sense of utility. Interestingly though, the EMA allowed us to detect that half of the sample did present alarms associated with lapses. In total, 45.5% of the participants reported a mean of two lapses and a range that ranged from 1 to 4, with a mean duration of approximately 3 h and an average money spent of 351.11 € (*SD* = 388.74; *range* = 71.53 – 1020.02).The intention of this EMA and the subsequent EMI system was to motivate patients to remain abstinent, or in the event of a lapse, to improve their awareness of the factors that may have affected them, to be able to implement effective strategies in the future in similar risky situations. Future studies could focus on studying the extent to which the intervention affects the motivation to remain abstinent and other gambling-related variables (e.g., gambling urges) in more detail.

Finally, regarding the preliminary results of treatment efficacy, there were significant improvements in the gambling urges and the perceived self-efficacy to cope with gambling urges from pre-treatment to post-module 3. Gambling urges decreased while participants perceived themselves as more capable of dealing with gambling-related high-risk situations. In addition, we also found a non-significant tendency of the participants to improve their anxiety (OASIS; Campbell-Sills et al., 2009; González-Robles et al., 2018) and depressive symptomatology (ODSIS; Bentley et al., 2014; Mira, González-Robles et al., 2019). These are encouraging preliminary results that are in the same line as Hawker et al. (2021), who reported reductions in the average number of gambling episodes, the intensity of gambling urges and frequency, and a rise in gambling self-efficacy over the intervention period using an internet program.

This study has some limitations. First, the sample size was small and we report descriptive preliminary results about the feasibility and preliminary effectiveness of this program concerning only the first three modules. Therefore, these results should be considered with caution. Nevertheless, this study makes substantial contributions by demonstrating the feasibility of continuing this research line, particularly with the involvement of local associations or clinics. The study also showed preliminary evidence regarding the program’s utility. However, it also evidenced the need to adapt the program’s conditions, such as increasing time per module to two weeks approximately and extending the duration of the weekly phone calls when lapses occur. In addition, it is crucial to consider increased therapeutic support, such as proposing a blended format. The online program could be combined with in-person or videoconference group sessions while utilizing EMA/EMI to monitor participants' progress. According to Burlingame et al. (2018), participating in group sessions offers numerous advantages, including opportunities for interpersonal connections, individuality, collaboration, and team dynamics among participants. It also provides individuals with support through shared knowledge and experiences with their peers. Additionally, internet-delivered treatments retain their benefits, such as enhanced accessibility and cost-effectiveness, as they require fewer resources compared to traditional treatments. Although research on blended treatments is still scarce due to their novelty and feasibility, there are still some efficacy results being obtained for the treatment of emotional disorders (Bielinski et al., 2020) and smoking cessation (Choi & Kim, 2018; Siemer et al., 2020). Another limitation refers to the assessment instruments used. In this study, some of them, such as the OASIS and the NODS, showed weak internal consistency, which may be influenced by the sample size and the low variability of the responses. However, previous studies show that they do have adequate psychometric properties (González-Robles et al., 2018; Hodgins, 2004). Likewise, for future research studies, the use of the NODS based on the DSM-5 criteria will be considered, as it has good psychometric properties (Brazeau & Hodgins, 2022). In addition, regarding the limitations of the instruments used, these consisted of self-report measures only. Thus, response bias could be present, which is relevant considering the characteristics of some individuals with GD, who sometimes omit and lie about their gambling behavior to others, as mentioned in the DSM-5 gambling disorder criteria. Thus, although co-therapists are considered in the treatment process to support the patients on the main therapeutic components (e.g., control stimulus and exposure with response prevention), it would be interesting to consider their participation in the EMAs to contrast the self-report information. It would also be interesting to incorporate greater involvement and contact with co-therapists, if possible, allowing them to better understand this issue and how to manage specific situations. This could potentially increase treatment adherence among participants as well. The EMA questions are based on what has happened in the last 24 h. Although it is an assessment of very recent states or behaviors this could still affect the presence of retrospection bias which should be taken into account. Future studies could address these limitations by using event-based or randomly scheduled time-based recordings. However, it’s important to avoid excessive demands on participants to prevent burnout, which was one of the reasons why we only established one measurement time. Another suggestion is the inclusion of more rigorous systems, such as the use of location-based technologies, which would allow evaluation when it detects that individuals are approaching pre-established risk zones.

Finally, the sample was composed of Spanish, Mexican, and Colombian participants who were residing in their countries, except one Colombian participant who lived in the USA. Cultural differences were found such as variations in the language and expressions, in the examples of gambling places, and also in the strategy of self-exclusion included in the stimulus control module. It was necessary to give them similar guidelines within their possibilities given that it was not possible to do self-exclusion as it is done in Spain, as reported by the participants (e.g., resorting to self-exclusion from specific web pages, specific face-to-face venues). Despite these limitations, they were able to follow the program with adequate preliminary results in terms of gambling variables. Ethically, these were people who had a high severity and needed immediate help, and who had difficulties receiving other types of treatment, due to economic limitations and geographical barriers. However, future studies could suggest adjustments for these countries to advance in this research line and better help individuals who suffer from this problem and need help, following, for instance, the guidelines of Salamanca-Sanabria et al., (2019, 2020).

Despite these shortcomings and the recruitment and reach difficulties, likely due to the complexity of GD, the external and internal difficulties in seeking help, and the technological profile of individuals with GD, the current online treatment shows promising preliminary data regarding its excellent appropriateness and usability. Moreover, it offers preliminary data on its generally acceptable adherence and potential utility to reduce gambling symptomatology in people from different Spanish-speaking countries who could not receive help in another way due to geographical barriers, time limitations, and lack of resources, among others. To improve treatment adherence, it may be beneficial to explore modifications such as transitioning to a blended group format and considering the involvement of co-therapists in future research endeavors.

## Supplementary Information

Below is the link to the electronic supplementary material.

Supplementary file1 (DOCX 794 KB)

## Data Availability

Data will be made available upon reasonable request. For research purposes or if clinical centers are interested in consulting the content of the modules, we will send them temporary links that last 30 days upon demand.
