# Supplementary material for: A Self-Applied Psychological Treatment for Gambling-Related Problems via The Internet: A Pilot, Feasibility Study
Source: J Gambl Stud. 2024 May 25;40(3):1623–51. doi: 10.1007/s10899-024-10318-2 (PMC11390850; doi:10.1007/s10899-024-10318-2)
Supplement: Supplementary file 1 — Supplementary file1 (DOCX 794 KB) [file 10899_2024_10318_MOESM1_ESM.docx]

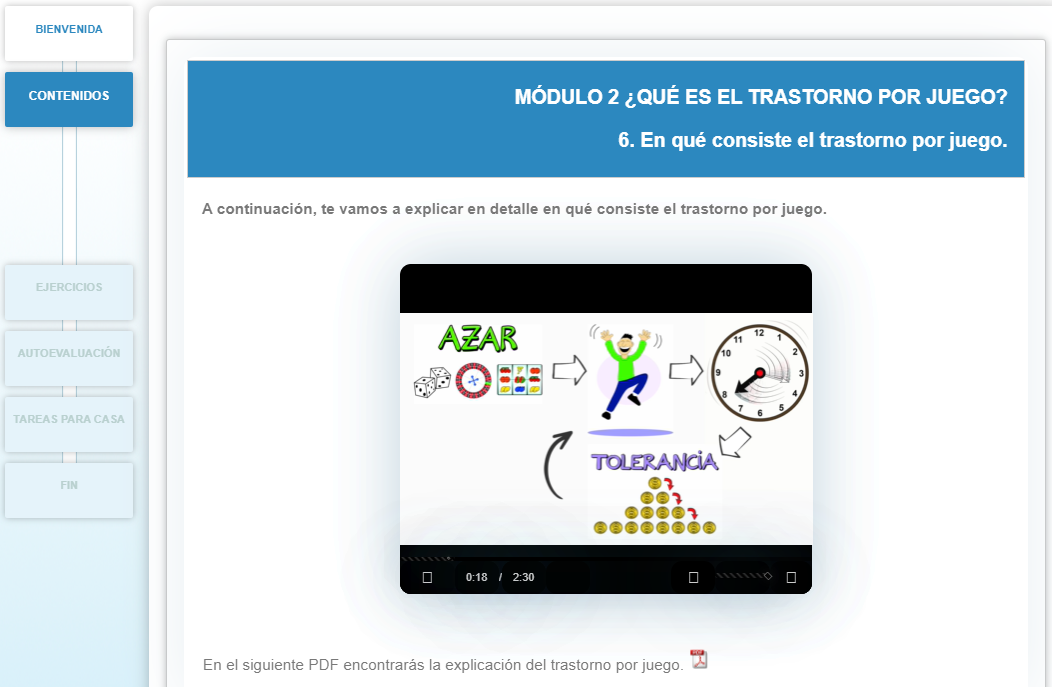


Screenshot of the content structure of the ‘Psicología y Tecnología’ [Psychology and Technology] web platform.


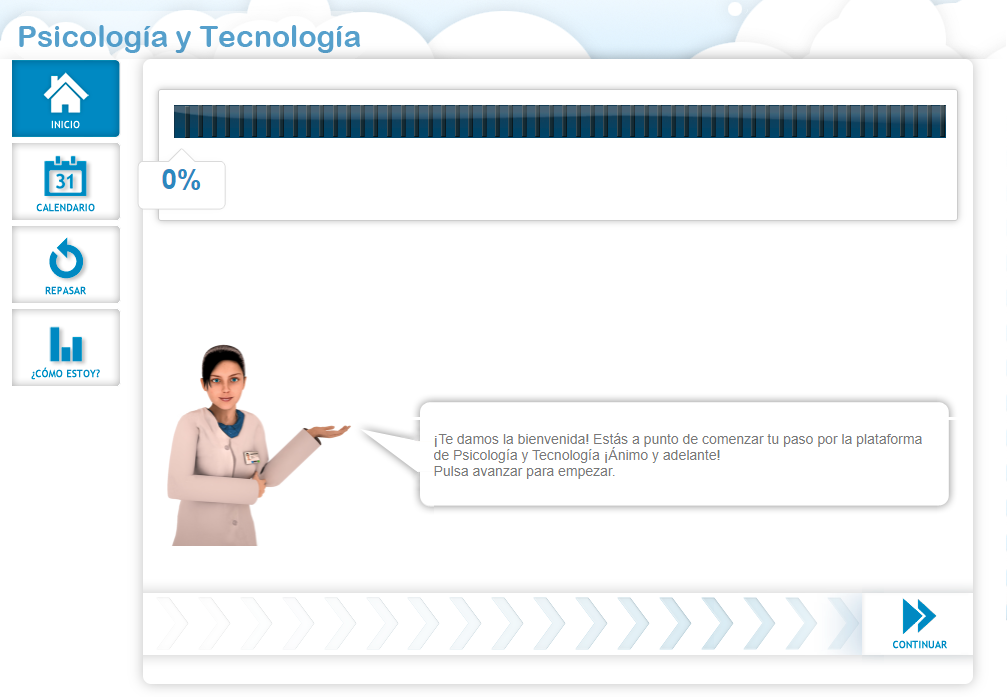


Screenshot of the ‘home ’complementary tool of the ‘Psicología y Tecnología’ [Psychology and Technology] web platform.


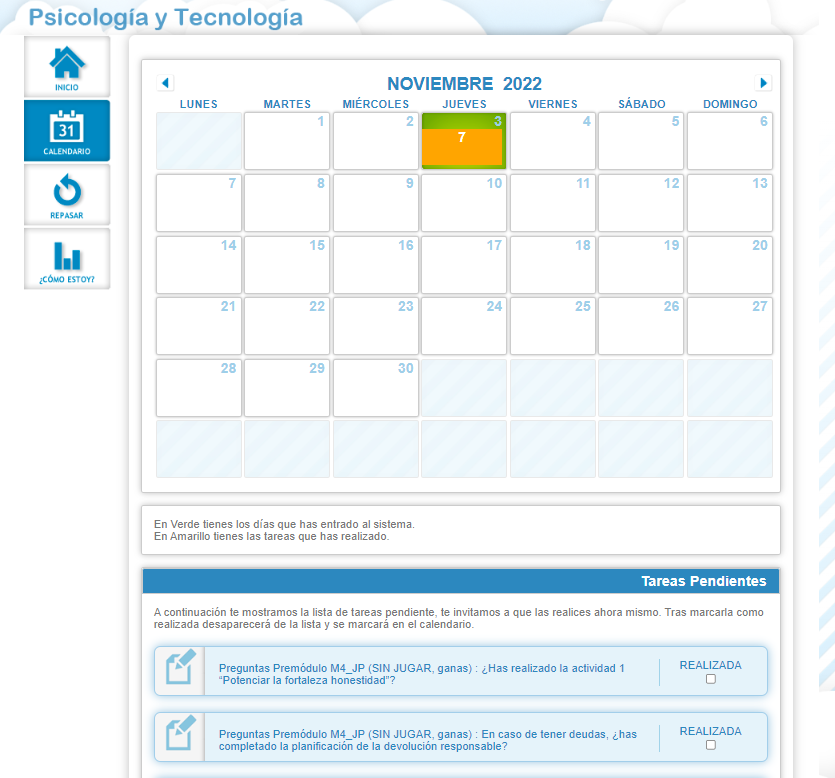


Screenshot of the ‘calendar ’complementary tool of the ‘Psicología y Tecnología’ [Psychology and Technology] web platform.


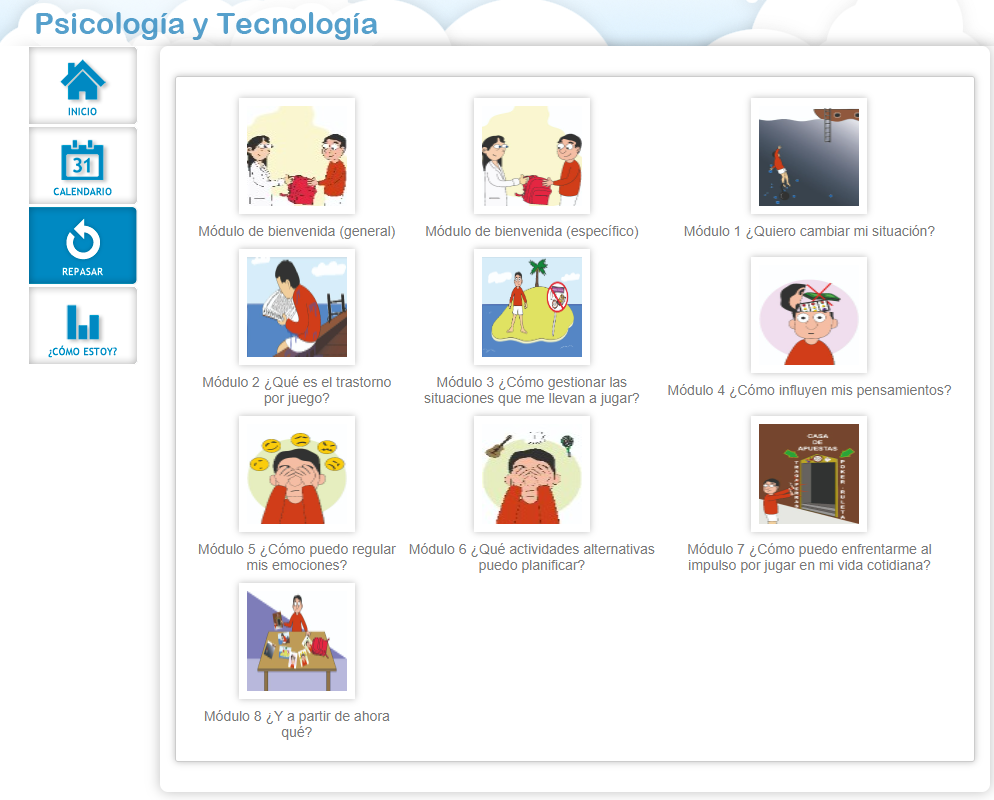


Screenshot of the ‘What have I learned’? complementary tool of the ‘Psicología y Tecnología’ [Psychology and Technology] web platform.


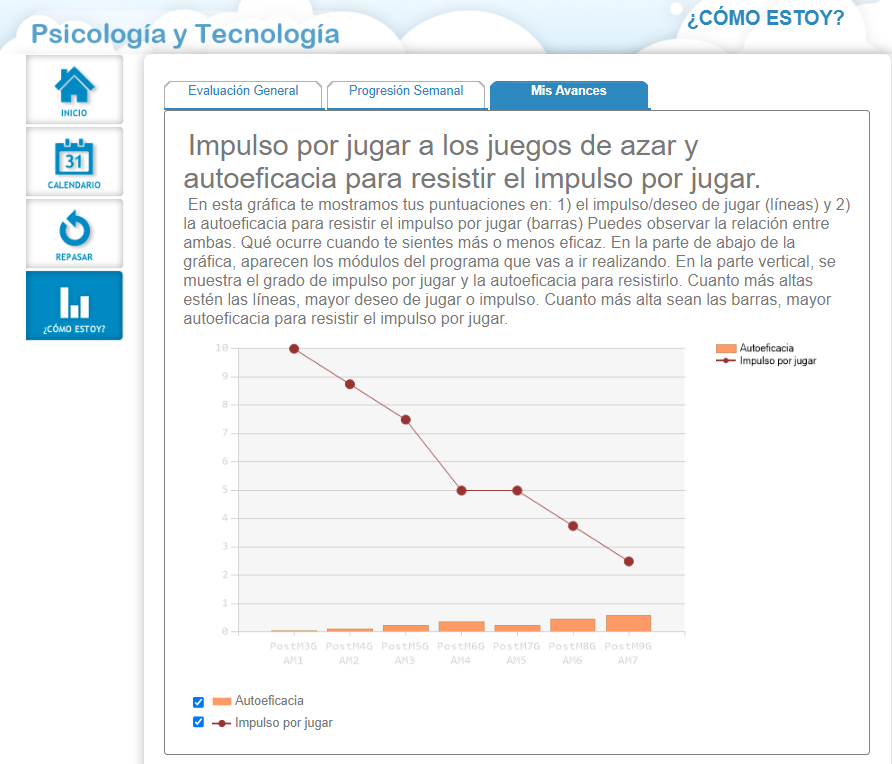


Screenshot of the ‘my progress’ complementary tool of the ‘Psicología y Tecnología’ [Psychology and Technology] web platform.
